# The association between socioeconomic status and pandemic influenza: protocol for a systematic review and meta-analysis

**DOI:** 10.1186/s13643-018-0931-2

**Published:** 2019-01-04

**Authors:** Svenn-Erik Mamelund, Clare Shelley-Egan, Ole Rogeberg

**Affiliations:** 1 Work Research Institute at OsloMet - Oslo Metropolitan University, PO. Box 4, St. Olavs plass, 0130 Oslo, Norway; 2 Frisch Centre, Gaustadalleen 21, 0349 Oslo, Norway

**Keywords:** Pandemic influenza, Morbidity, Hospitalization, Mortality, Socioeconomic status, Education, Income, Poverty, Occupational social class, Housing conditions

## Abstract

**Background:**

Pandemic mortality rates in 1918 and in 2009 were highest among those with the lowest socioeconomic status (SES). Despite this, low SES groups are not included in the list of groups prioritized for pandemic vaccination, and the ambition to reduce social inequality in health does not feature in international and national pandemic preparedness plans. We describe plans for a systematic review and meta-analysis of the association between SES and pandemic outcomes during the last five pandemics.

**Method:**

The planned review will cover studies of pandemic influenza that report associations between morbidity, hospitalization, or mortality with socioeconomic factors such as education and income. The review will include published studies in the English, Danish, Norwegian, and Swedish languages, regardless of geographical location. Relevant records were identified through systematic literature searches in MEDLINE, Embase, Cinahl, SocIndex, Scopus, and Web of Science. Reference lists of relevant known studies will be screened and experts in the field consulted in order to identify other additional sources. Two investigators will independently screen and select studies, and discrepancies will be resolved through discussion until consensus is reached. Covidence will be used. Results will be summarized narratively and using three meta-analytic strategies: coefficients expressing the difference between the highest and lowest socioeconomic groups reported will be pooled using (a) fixed and random effects meta-analysis where studies involve similar outcome and exposure measures and (b) meta-regression where studies involve similar outcome measures. In addition, we will attempt to use all reported estimates for SES differences in (c) a Bayesian meta-analysis to estimate the underlying SES gradient and how it differs by outcome and exposure measure.

**Discussion:**

This study will provide the first systematic review of research on the relation between SES and pandemic outcomes. The findings will be relevant for health policy in helping to assess whether people of low socioeconomic status should be prioritized for vaccines in preparedness plans for pandemic influenza. The review will also contribute to the research literature by providing pooled estimates of effect sizes as inputs into power calculations of future studies.

**Systematic review registration:**

PROSPERO 87922

## Introduction

Reducing social inequality in health is a core aim of international policies and health work, but a recent review of international and national pandemic preparedness plans finds this perspective lacking in this policy area [1]. No country currently includes low socioeconomic status (SES) groups on their list of groups prioritized for pandemic vaccination. This is surprising given that pandemic mortality rates both in 1918 and in 2009 are highest among those with the lowest SES.

To our knowledge, our study will be the first systematic review and meta-analysis on the associations between SES and pandemic outcomes. A systematic review has been carried out on the associations between disadvantaged ethnic populations and pandemic outcomes in 2009 [2], and a few countries have put ethnic minorities on their list of risk groups for vaccination (e.g., the USA, Canada, and Australia). Our research will provide a strong foundation for assessing whether low SES groups should be placed on the recommended lists of pandemic vaccination along with high-risk age groups, the pregnant, ethnic minorities, health care workers, and the previously ill.

Our research question is whether—and to what extent—socioeconomic status measured by covariates such as education or income is associated with pandemic morbidity, hospitalization, or death. In terms of PICO criteria, the Population (P) consists of groups defined by socioeconomic status, the intervention (I) or exposure or risk factor is pandemic influenza, the comparison (C) or alternative interventions is not relevant, while the outcomes (O) are morbidity, hospitalization, or death associated with influenza pandemics.

A number of studies suggest that pandemic outcomes vary with income and socioeconomic status. During the 1918 pandemic, mortality rates were reduced in high-income countries relative to low-income countries and for rich individuals relative to poor in towns with a large degree of social inequality at baseline. For instance, India had a mortality rate 40 times higher than Denmark [3], while research on Norway found raised mortality rates for members of the working class and those living in small flats and on the east side of the capital city Oslo [4]. Recent studies have also demonstrated that the illiterate, the unemployed, and those renting their homes suffered higher mortality than their counterparts in the city of Chicago, [5] and in Sweden, there was a clear occupational class gradient in mortality [6]. By contrast, countries with small social inequalities at baseline, such as New Zealand, had no mortality differences by socioeconomic status [7–9]. During the 2009 pandemic, some South American countries had a mortality rate 20 times that of Europe [10], while poorer parts of England had mortality rates three times those of more affluent parts [11].

Similar results have been reported for other severe outcomes from pandemics, such as hospitalizations [12, 13], although studies of less severe outcomes such as lab-confirmed H1N1 infection rates during the 2009 pandemic did not appear to differ with socioeconomic indexes across areas in Brisbane, Australia, or by neighborhood socioeconomic status in French administrative regions [14, 15].

Although the studies referred to above report social inequalities in severe outcomes in 1918 and in 2009, more studies are needed to uncover and separate the distal (social and policy) and proximal (behavioral and biological) risk factors for unequal exposure, susceptibility, and access to health care leading to unequal pandemic outcomes [16]. These may relate to cramped living conditions, occupational exposure, ability to stay away from work in order to care for family members, poor nutritional status, concurrent illnesses, and a lack of understanding of or access to health advice (e.g., hand hygiene) and vaccination recommendations due to poor reading and writing skills [1, 4].

Based on the above studies and theoretical arguments for why SES might be dependent on the severity of the pandemic outcome [4], we hypothesize that the association between SES and pandemic outcomes increases with outcome severity, for instance, because higher income and SES are associated with access to resources and protective factors that reduce the risk of progression to more severe outcomes. To test this hypothesis, we will carry out a systematic review and meta-analysis of the literature on the associations between socioeconomic status and pandemic outcomes in the last five pandemics. These are the Russian influenza in 1889–90: Spanish Influenza in 1918–1920, Asian influenza of 1957–1958, the Hong Kong flu of 1968–1970, and the swine flu of 2009–2010.

Although influenza pandemics have occurred three to four times each century since the first generally recognized pandemic occurred in 1580 [17], our analysis concentrate on pandemics that occurred in the period 1889–2009, for three reasons. First, because the detailed sociodemographic data needed to analyze the topic of interest do not exist or are scant for most countries prior to the 1889–1890 pandemic, few, if any, have studied the role of SES for pandemics prior to 1889. Second, the farther we go back in history, the less likely is it that experiences during historical pandemics are relevant and can be translated into current and future influenza pandemic preparedness. This may be an issue even within our covered period of some 120 years, a possibility that will be examined through subsample analyses (see below). Even within our covered period, the results from recent pandemics may differ from those occurring 100–120 years ago. Third, by studying the five last pandemics, our systematic review will cover the breadth of the topic by geographic and pathogen variety over time that cannot be captured by studying, for example, only the 2009 pandemic. We expect to find most studies focused on the 2009 pandemic, followed by the 1918 pandemic, and with very few studies on the pandemics of 1957–1958 and 1968–1970.

## Methods/design

### Bibliographic database search

A comprehensive and exhaustive search of MEDLINE, Embase, Cinahl, SocIndex, Scopus, and Web of Science was performed to identify all relevant articles published on socioeconomic factors and pandemic influenza. The strategy for the literature search was developed by two information specialists in cooperation with the research group. Several pilot searches were conducted in MEDLINE and Web of Science to ensure a sensitive search. The search strategy combines relevant terms, both controlled vocabulary terms (i.e., MeSH) and text words. The main search strategy used in MEDLINE is available in PROSPERO 87922. The strategy was translated and modified to fit the other databases listed above. To generate manageable results, restrictions on language (English, Danish, Norwegian, and Swedish) and publication type (article/research article) were added to the searches in the other databases. The searches in MEDLINE and Embase were performed without publication type restrictions. The search strategy was peer-reviewed by a third information specialist using a structured tool based on the PRESS framework [18]. In order to identify further relevant books not covered by the databases used in this review, we will ask experts in the field for additional research titles and to search the reference lists of the most relevant articles for additional sources.

### Inclusion criteria for title and abstract screening

We identified 8411 records through database searching. After a preliminary elimination of duplicates (*n* = 4202), the results from the Endnote library were imported to the program Covidence (*n* = 4203). Here, additional duplicates were discovered and removed (*n* = 78), leaving 4128 hits. Each article’s title and abstract will be screened by the two authors (SEM and CSE), according to the selection criteria. After the screening of titles and abstracts, full-text versions will be added in Covidence. Divergences in the inclusion of studies will be re-assessed by the same researchers until consensus is reached in terms of inclusion or exclusion. The criteria for inclusion are as follows:

1. The study period 1889–2009 including the five pandemics in 1889, 1918, 1957, 1968, and 2009
2. Studies looking at the quantitative associations between SES on the one hand, and morbidity, severe disease, and mortality from pandemic influenza, on the other. SES is captured by keywords such as education, income, and occupational social class (see search history for more examples). Morbidity is captured by keywords such as infection rates, transmission rates, lab-confirmed influenza, flu-like illness, and influenza-like illness (ILI). Severe disease is captured by keywords such as disease severity, critical illness, critical disease, severe illness, severe disease, hospitalization, patient admission, hospital admission, intensive care unit (ICU) admission, and ICU treatment. Mortality is captured by keywords such as fatal outcome, fatal illness, fatal disease, fatality, lethal outcome, lethal illness, lethal disease, terminal outcome, terminal illness, terminal disease, lethality, death, death rate, and mortality rate. All of these keywords were used in both pilots and the final search as described above. The search strategy also covered studies of ethnic and disadvantaged populations, as we expect some of these to include covariates for socioeconomic confounders that would fall within our inclusion criteria
3. Studies covering both seasonal and pandemic influenza separating between non-pandemic and pandemic years
4. Studies covering all regions/countries, type of studies (interventional, observational, etc.), and populations (age, gender, pregnant women, soldiers, etc.)

### Exclusion criteria for title and abstract screening

The following criteria will exclude studies from the systematic literature review:

1. Studies on other pandemic diseases than influenza
2. Studies on seasonal influenza only
3. Studies on both seasonal and pandemic influenza that *do not* separate between non-pandemic and pandemic years
4. Studies on influenza vaccine uptake, attitudes towards influenza vaccination, and compliance with (non)pharmaceutical interventions during influenza pandemics
5. Case studies or qualitative studies on the associations between socioeconomic factors and pandemic outcomes
6. Studies on social justice and pandemic influenza
7. Studies of pandemic influenza preparedness plans
8. Studies on ethnic and disadvantaged minorities that *do not* report controls for socioeconomic confounders

### Data selection and extraction

We will draft a data abstraction form, pilot test it and modify it, if necessary. Two reviewers (SEM and CSE) will independently extract data from all included studies. Any disagreements will be resolved via discussion or by involving a third reviewer for arbitration; 1–5 and 6 below will be entered into separate spreadsheets for each article. The following information will be extracted:

1. Article info
  - First author
  - Year published
  - Journal
2. Data sample
  - Country or region of analysis
  - Pandemic years (1889, 1918, 1957, 1968, 2009)
  - Sample inclusion criteria—i.e., characteristics of sample/population (civilian, military, gender, pregnant, age-group/median/average age, patient group, etc.)
  - Sample size
  - Unit of analysis (individuals, households, regions, hospitals, etc.)
  - Data aggregation level (observations of individual units, aggregated units, etc.), e.g., if hospitals are the unit of analysis, does the data used occur at the hospital level or is it pooled across hospitals?
  - Source of outcome data, e.g., census, routine notification data (e.g., influenza cases reported to a doctor), survey data, register data
    i. If survey or incomplete coverage of population data
      1. Response rate/coverage
      2. Is the sample shown to be representative for the population? That is, has a non-response analysis been carried out?
3. Outcome variable—pandemic outcome ((a) morbidity, (b) hospitalization, (c) mortality)
  - Give the definition of morbidity: ILI, lab-confirmed infection rates (PCR), transmission rates (reproduction number, R0), immunity/antibodies towards influenza (HI titer above a certain threshold) etc.
  - Give the definition of hospitalization: hospitalized inpatients with (PCR) or without confirmed influenza, patients admitted to intensive care unit (ICU) or not, mechanically ventilated patients (“lung machines”) or not, inpatients vs outpatients) etc.
  - Give the definition of cause of mortality: influenza and pneumonia (PI), excess mortality (PI, all causes of death, etc.), respiratory diseases, pneumonia, etc.
4. Independent variables of interest—relating to SES
  - Type of SES indices (education, income, crowding, density, deprivation index, unemployment, occupational social class, poverty status, % below poverty level)
  - Give definition or brief descriptive text on SES indices (e.g., if based on a specific type of poverty index, etc.)
5. Statistical methodology
  - Design of study (cross-sectional, longitudinal, case-control, cohort studies)
  - Estimation technique (cross tables, correlation analysis, OLS, Poisson regression, logistic regression, Cox regressions, GEE regressions, GLMM models, etc.)
  - Report all control variables (e.g., age, gender, marital status, pre-existing disease, health behavior, etc.) in light of sample restrictions (e.g., for pregnant women, sex is not among the controls)
  - Report on the reference categories with which all point estimates are compared
6. Results reported (separate spreadsheet)

### Quality assessment

We will use the *Newcastle Ottawa Scale* (NOS) to evaluate the quality of the included studies. NOS can be used to evaluate the risk of bias in nonrandomized studies such as case-control and cohort studies and consist of three domains: selection, comparability, and exposure [19].

We will score studies on the representativeness of each sample/data source, whether studies control for important confounders (e.g., age, gender, baseline health etc.) or not, or whether studies are using aggregate- or individual-level data. For instance, a representative sample using individual data and a broad set of relevant controls will receive higher quality ratings than studies using aggregate data, narrow samples, or lacking important confounding variables.

### Data synthesis

We will synthesize our results both narratively and quantitatively. The narrative (descriptive) review will include a table of the study characteristics of the included studies, such as author, year, pandemic years, region/country/hospital, sample size and type of data, population by type of socioeconomic status indices (*N* and %), and pandemic outcome (1–5 in data extraction protocol above). Missing data will be requested from authors, e.g., number of events and population at risk, and quality will be assessed using the NOS. The presence of publication bias will be assessed using funnel plots and the Egger test, as well as through the *p* value of the standard-error covariate in the PET-PEESE meta-regression (see below).

The quantitative part of the study will pool results across studies. Such pooling can be done using various methods that impose different constraints on the type of studies that can be pooled. We will pursue three strategies. The first two are within the frequentist statistical tradition. We will here note whether coefficients are statistically significant at the 1%, 5%, or 10% level, i.e., whether the evidence base indicates pooled effects that would be unlikely if the true effect was zero. We will also discuss the strength of this test by assessing the magnitude of the pooled coefficient and its standard error (precision) in relation to plausible effect sizes. In the third, Bayesian methods will be used and we will assess how the evidence updates weakly informative priors for the coefficients.

#### Pooled effect meta-analysis

Where several studies are available with similar outcome and exposure measures, we will show forest plots and estimate pooled effects using fixed and random effects analyses with the metafor meta-analytic package in R [20], transforming the outcome variable when this is required to make the sampling distribution approximate the normal distribution, e.g., taking the log of odds ratios or using the Freeman-Tukey double arcsine transformation for proportions. In these analyses, the pooled estimates will reflect comparisons of the highest to the lowest reported socioeconomic group. We expect random effects to be more appropriate, since the socioeconomic gradient in outcomes may differ across time and region (e.g., we would expect a lower gradient in countries and periods with lower inequality). Cochran’s *Q* test will be used to assess whether data indicate statistically significant heterogeneity in effects at the 5% level. Effect estimates are also expected to differ systematically across studies according to the socioeconomic “distance” between compared groups. For instance, we would expect a larger outcome difference between the top and bottom 10% of a distribution than we would between those above and below the mean. Depending on the total number of studies that can be pooled in a given analysis, it may also be appropriate to conduct subsample analyses that assess whether pooled effects differ within subgroups of studies characterized by region, pandemic, age-group, gender, and estimation technique or quality assessment score.

#### Meta-regression

A recent innovation in meta-analyses is a meta-regression technique with precision effect test and precision effect estimate with standard error (“PET-PEESE”) [21, 22]. This technique will be used to pool estimates with similar outcome measures and will allow us to include study-level information as covariates and explore how these correlate with the coefficient estimates. This allows us to assess whether coefficients from comparisons of educational groups tend to differ from those comparing income groups, whether coefficients vary systematically by study-level variables such as pandemic, country-level inequality measures, statistical methodology used, or quality assessment score. The method additionally allows for the examination of how estimates differ systematically with e.g., age-groups. Finally, the technique tests whether coefficients vary systematically with reported standard errors, which may indicate the presence of small sample or publication bias.

#### Bayesian meta-analysis

The above strategies require a similar outcome measure and will pool coefficients for the highest relative to the lowest socioeconomic group from each study. This ignores the “dose-response” information available from studies that report coefficients comparing multiple socioeconomic levels to a reference level (e.g., coefficients for different income quantiles). Under the assumption that an underlying socioeconomic gradient will be linear on the logit scale, all such reported estimates can contribute to estimating the underlying gradient [23, 24]. The resulting statistical model will be coded and estimated using the Stan language for probabilistic modeling [25] with a multilevel/hierarchical specification to account for heterogeneity across exposure measures (e.g, income, education), pandemic, and study-level covariates. We will also explore whether such an approach makes it feasible to pool studies across outcome measures to assess the hypothesis that gradients vary systematically by the severity of outcome.

## Discussion

To the best of our knowledge, our review will be the first to review systematically the evidence for a link between socioeconomic status and pandemic outcomes. The review will generate insights for health policy. If socioeconomic risk factors are shown to be important in explaining the variation in pandemic outcomes above and beyond biomedical risk factors, influenza pandemic preparedness plans should include discussion regarding how to reduce social inequalities in pandemic outcomes, e.g., by recommending vaccination to certain households with below poverty income or people living in a designated poverty area. If socioeconomic risk factors cannot explain the association between the traditional biomedical risk factors and pandemic outcomes, pandemic preparedness plans do not need to be changed. However, we still need to address the underlying reasons for inequalities in health and fight poverty as part of UN Sustainable Development Goals.

## Abbreviations

CSE: Clare Shelley-Egan
OR: Ole Rogeberg
SEM: Svenn-Erik Mamelund
SES: Socioeconomic status
